# Effect of boundary conditions on the evolution of lattice strains in a polycrystalline austenitic stainless steel

**DOI:** 10.1007/s10853-017-0997-6

**Published:** 2017-03-17

**Authors:** Y. Q. Wang, S. Hossain, S. Kabra, S. Y. Zhang, D. J. Smith, C. E. Truman

**Affiliations:** 1 Department of Mechanical Engineering, University of Bristol, Bristol, BS8 1TR UK; 2 School of Materials, The University of Manchester, Oxford Rd, Manchester, M13 9PL UK; 3 MXIF, Research Complex at Harwell, Harwell Campus, Oxfordshire, OX11 0FA UK; 4 Department of Aeronautical Engineering, Military Technological College, Muscat, Oman; 5 ISIS Facility, STFC, Rutherford Appleton Laboratory, Didcot, Oxfordshire, OX11 0QX UK

## Abstract

The effect of boundary conditions (constant load, constant strain and elastic follow-up) on lattice strain evolution during creep in a polycrystalline austenitic stainless steel was studied using in situ neutron diffraction at 550 °C. The lattice strains were found to remain constant under constant load control. However, under constant strain and elastic follow-up control, the lattice strains relaxed the most in the elastically softest lattice plane {200} and the least in the elastically stiffest lattice plane {111}. The intergranular stresses created between different grain families were constant during creep tests irrespective of the boundary conditions with the initial applied stresses of 250 MPa.

## Introduction

**Figure 1:**
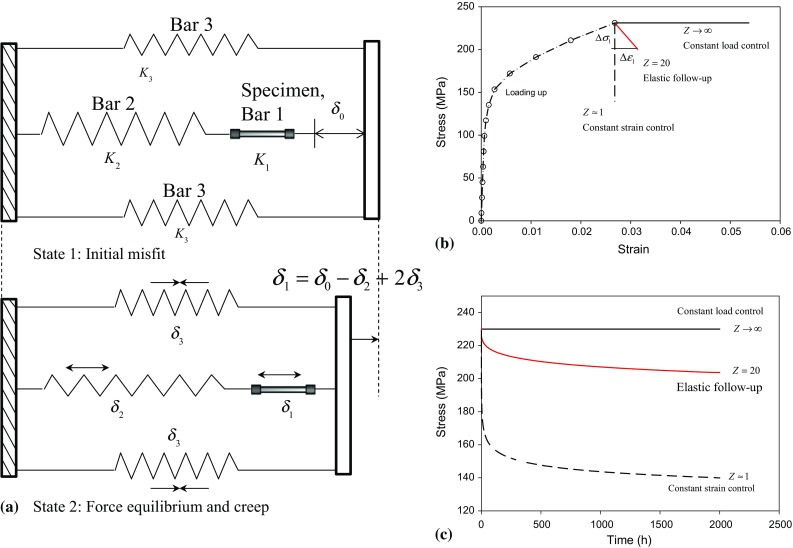
Schematic diagrams illustrating a three-*bar* system and the mechanical behaviour of a test specimen due to creep; **a** the three-*bar* system allows the introduction of misfit, and the system provides elastic follow-up during creep stress relaxation; **b** the stress–strain trajectories for loading up and creep at constant load creep ($Z \to \infty$), elastic follow-up ($1 < Z < \infty$) and stress relaxation ($Z = 1$); and **c** stress relaxation *curves* with elastic follow-up factor equal to 1 and 20, respectively

The term elastic follow-up was first introduced by Robinson [1] to explain the relaxation of bolted joints due to creep. Wang et al. [2] developed a three-bar system to generate a tensile stress in a specimen (bar 1) through the introduction of a misfit ($\delta_{1} = \delta_{0} - \delta_{2} + 2\delta_{3}$), as shown in Fig. 1a, where $\delta_{0}$, $\delta_{1}$, $\delta_{2}$ and $\delta_{3}$ are the total misfit, misfits in the specimen, bar 2 and bars 3, respectively. When the stress in the specimen relaxes due to creep, the stress and deformation in bars 2/3 decrease/increase and tend towards their unloaded position. This results in an increase in the deformation of the specimen. This displacement redistribution is called the elastic follow-up for creep which can exist in many engineering components operating at high temperature [3]. When the specimen experiences creep while the remaining bars are elastic [4, 5], an elastic follow-up factor, *Z*, is given by.

1 $$ Z = 1 + \frac{1}{\beta } + \frac{1}{\gamma } $$

where $\beta$ and $\gamma$ are the stiffness ratios between the specimen and the bars 2 and 3, given by $\beta = \frac{{K_{2} }}{{K_{1} }}$ and $\gamma = \frac{{2K_{3} }}{{K_{1} }}$, where $K_{1} = \frac{{A_{1} E_{1} }}{{L_{1} }}$, $K_{2} = \frac{{A_{2} E_{2} }}{{L_{2} }}$ and $K_{3} = \frac{{A_{3} E_{3} }}{{L_{3} }}$ are stiffness for the specimen, bars 2 and 3, respectively. For each of the bars, $A$, $E$ and $L$ are the cross-sectional area, the Young’s modulus and the bar length, respectively.

Equation 1 shows that for large stiffness ratios $\beta$ and $\gamma$ the elastic follow-up factor *Z* tends to 1 and essentially represents constant strain control (stress relaxation). In contrast, very small stiffness ratios $\beta < < 1$ and $\gamma < < 1$ result in infinitely large elastic follow-up factor ($Z \to \infty$). This represents constant load-controlled boundary condition (forward creep). The boundary conditions in between the constant strain control and the constant load control are the elastic follow-up control. This range of behaviour is illustrated in Fig. 1b and shows the difference in the stress–strain curves for constant load, constant strain and elastic follow-up boundary conditions. The presence of elastic follow-up results in a slower stress relaxation rate (Fig. 1b, c) and additional strain accumulation in the specimen (Fig. 1b) when compared to a classical stress relaxation test.

It is well known that when a polycrystalline material undergoes macroscopic plastic deformation, intergranular strains or stresses can be generated within grain families as a consequence of elastic–plastic anisotropy at the grain scale [6–9]. Creep as a time-dependent plastic deformation can also generate intergranular strains/stresses in Type 316H austenitic stainless steel often during primary stage of the constant load creep [10–12]. This is due to creep occurring differently along different crystalline planes, thereby creating strain incompatibilities between grain families. The presence of intergranular stress can change the internal resistance and effective stress in materials which therefore change the material properties [13–16]. However, no work has been done to study the effect of elastic follow-up on creep behaviour along different crystalline orientations. The motivation for the current study arose from a need to understand and compare the evolution of intergranular strains and stresses during forward creep, stress relaxation and elastic follow-up. With such understanding, a new creep model can be built to predict the stress relaxation and elastic follow-up behaviour and account for the elastic follow-up into structural integrity assessment and life extension of UK’s advanced gas-cooled reactors (AGRs) [17].

## Materials and experiment

The material used in the present work was ex-service laboratory-aged (EXLA) Type 316H austenitic stainless steel, supplied by EDF-Energy. This 316H stainless steel was from header HYA 2D1/2 (cast 69431) that had been in service for approximately 65000 h in the temperature range of 763–803 K, followed by exposure to 823 K for 21000 h. The chemical composition of the EXLA Type 316H austenitic stainless steel is given in Table 1 [14].

**Table 1:** Chemical composition of ex-service laboratory-aged Type 316H austenitic stainless steel in weight percentage (wt %) [14]

C	Si	Mn	P	S	Cr	Mo	Ni	Co	B	Fe
0.06	0.4	1.98	0.021	0.014	17.17	2.19	11.83	0.10	0.005	Bal.

A rig was built based on the three-bar model (Fig. 1a) which enables the in situ tracking of lattice strain evolution in 316H austenitic stainless steel during creep under different boundary condition. The rig was commissioned at the ENGIN-X instrument, Rutherford Appleton Laboratory (RAL), ISIS neutron facility, UK [18, 19]. For the present experiments, two different elastic follow-up factors were obtained by using different sample dimensions. A small elastic follow-up factor (*Z* ~ 1.2) was obtained by using a long cylindrical specimen with length 150 mm, diameter 6 mm (stiffness *K*_*1*_ 28 kN mm^−1^) connected to the rigid rig frame. A larger elastic follow-up factor (*Z* ~ 10.5) was obtained by using a short specimen with length 30 mm, diameter 7 mm (stiffness *K*_*1*_ 192 kN mm^−1^) fitted in series to an aluminium round bar with length 250 mm, diameter 10 mm (stiffness *K*_*2*_ 19.6 kN mm^−1^). The elastic follow-up introduced by the remaining parts of the rig was negligible due to its large stiffness. A constant load control ($Z \to \infty$) experiment was also conducted using a tensile rig with a radiant air furnace at ENGIN-X.

For each test, the specimen was first heated to 550 °C. Load was then applied with strain rate of 0.0067% s^−1^, and the stress in the gauge length was increased in 25 MPa steps until the stress level of 200 MPa (*Z* = 1.2), 225 MPa (*Z* = 10.5) and 250 MPa ($Z \to \infty$) was reached. For *Z* = 1.2, for loading between 200 and 250 MPa, a slow loading up strain rate was used and a stress increment of about 3–5 MPa was applied every 5 min in order to avoid any significant stress decrease during measurement. For constant strain and elastic follow-up tests, the stepper motor was switched off once the measured stress of 250 MPa was achieved. The stress in the specimen decreased as the elastic strain converted to creep strain. For *Z* = 10.5, the specimen was reloaded to 350 MPa after 8-h relaxation from the initial applied stress of 256 MPa and further relaxed for about 13 h at 550°C. For the constant load creep test ($Z \to \infty$), the applied load was maintained at 250 MPa while the creep strain increased with time. These creep stages lasted around 8–25 h.

Elastic lattice strains along the axial direction in grain families having {111}, {200}, {220} and {311} crystallographic planes during loading up, creep and unloading were measured by neutron diffraction at approximately the middle position on the centreline of the test specimen using a 4 × 4 × 4 mm^3^ gauge volume. The acquired data were recorded over 10 and 5-min time spans during loading and creep stages, respectively. Changes in lattice spacing were used to calculate the internal strains using $\varepsilon_{hkl} = \frac{{d_{hkl} - d_{hkl}^{0} }}{{d_{hkl}^{0} }}$ where $\varepsilon_{hkl}$ is the elastic lattice strain in the $\left\{ {hkl} \right\}$ grain family, $d_{hkl}$ and $d_{hkl}^{0}$ are the sample lattice spacing and the stress-free lattice spacing at 550 °C, respectively. The new designed rig only allowed the diffracted neutrons collected by detector 1 with appropriate intensity, and data collected from detector 2 with reduced intensity were not used in the experiments. The uncertainty in the measured internal strains was approximately ±30 microstrain. The current materials can be assumed as texture-free polycrystalline [20].

## Results and discussion

**Figure 2:**
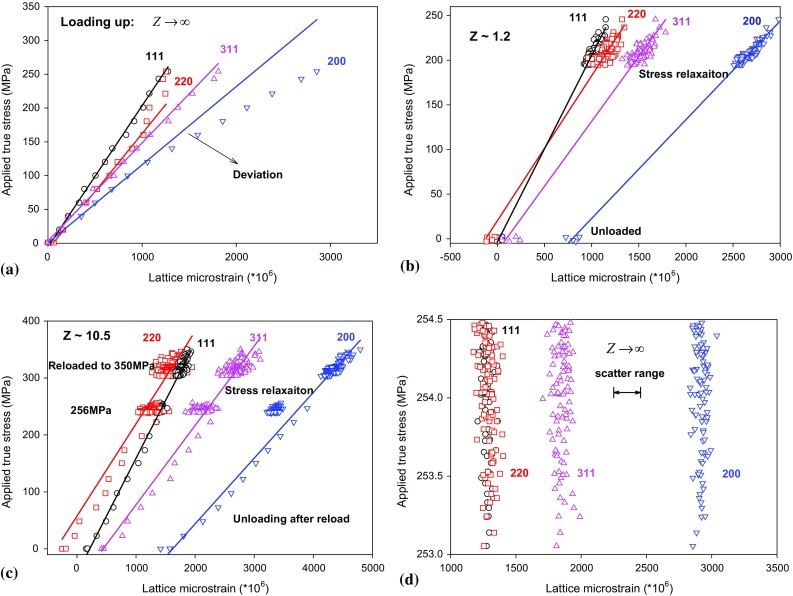
Neutron diffraction measured elastic lattice strains along the axial direction for {111}, {200}, {311} and {200} grain families during **a** loading for $Z \to \infty$; **b**–**c** creep and unloading stages for Z ~ 1.2 and Z ~ 10.5; and **d** creep stage for $Z \to \infty$

A typical example of applied true stress versus the lattice strain curve during loading up can be seen in Fig. 2a. All of the crystallographic planes deformed linearly at stresses lower than 120 MPa. A deviation in the linear response was observed at stresses greater than 120 MPa, which means some crystallographic planes started yielding. It should be noted that neutron diffraction always measures the elastic lattice strain. Yielded plane would not take up as much elastic strain (stress) as it would before yielding with increase in macroscopic stress. Here, as shown in Fig. 2a, the {220} plane yielded first and resulted in the {200} and {311} planes taking up the elastic strain (stress) redistributed from {220}. The elastic and plastic anisotropy at each plane caused strain incompatibilities and generated intergranular stresses between the grains at different orientations. The diffraction elastic constants (DEC) for each plane were obtained by dividing the change in applied stress by the corresponding change in lattice strain for each plane during unloading and are summarised in Table 2. The elastic moduli obtained in the current study perfectly agreed with the work conducted by Daymond and Bouchard [6]. The lattice strains relaxed with the relaxation of applied stresses linearly during creep under *Z* ~ 1.2 and *Z* ~ 10.5 and act similar to unloading process, as shown in Fig. 2b, c. Therefore, the lattice strains were found to relax the most in the elastically softest lattice plane {200} and the least in the elastically stiffest lattice plane {111} due to different grain families in crystalline materials displaying elastic anisotropies. The lattice strains remained constant with some degree of scatter under constant load control (Fig. 2d).

**Table 2:** Summary of derived diffraction elastic constants (DEC) from unloading of the in situ neutron diffraction measurements at 550°C

Test	$E_{111}$, GPa	$E_{200}$, GPa	$E_{220}$, GPa	$E_{311}$, GPa
Z ~ 1.2, unloading up	207	111	166	145
Z ~ 10.5, second unloading up	183	107	162	126
$Z \to \infty$, unloading up	195	116	163	140

The axial principal stress can be calculated by the strain vectors measured from neutron diffraction through the generalised Hooke’s law:

2 $$ \sigma_{{_{hkl} }}^{zz} = \frac{{E_{hkl} }}{1 + \upsilon }\varepsilon_{{_{hkl} }}^{zz} + \frac{{{{E_{hkl} } \mathord{\left/ {\vphantom {{E_{hkl} } \upsilon }} \right. \kern-0pt} \upsilon }}}{{\left( {1 + \upsilon } \right)\left( {1 - 2\upsilon } \right)}}\left( {\varepsilon_{{_{hkl} }}^{zz} + \varepsilon_{{_{hkl} }}^{\theta \theta } + \varepsilon_{{_{hkl} }}^{rr} } \right) $$

where the superscripts $zz$, $\theta \theta$ and $rr$ represent axial, hoop and radial principal directions in the cylindrical coordinate system, $\upsilon$ is the Poisson’s ratio. In the present study, the axial stresses for each plane were calculated assuming both the hoop strain ($\varepsilon_{{_{hkl} }}^{\theta \theta }$) and the radial strain ($\varepsilon_{{_{hkl} }}^{rr}$) were equal to $- \upsilon \varepsilon_{{_{hkl} }}^{zz}$ [14]. Hence, Eq. 2 reduces to

3 $$ \sigma_{{_{hkl} }}^{zz} = E_{hkl} \varepsilon_{{_{hkl} }}^{zz} $$

The lattice stresses were obtained by taking the product of the lattice strain and the corresponding DEC (Eq. 3).

**Figure 3:**
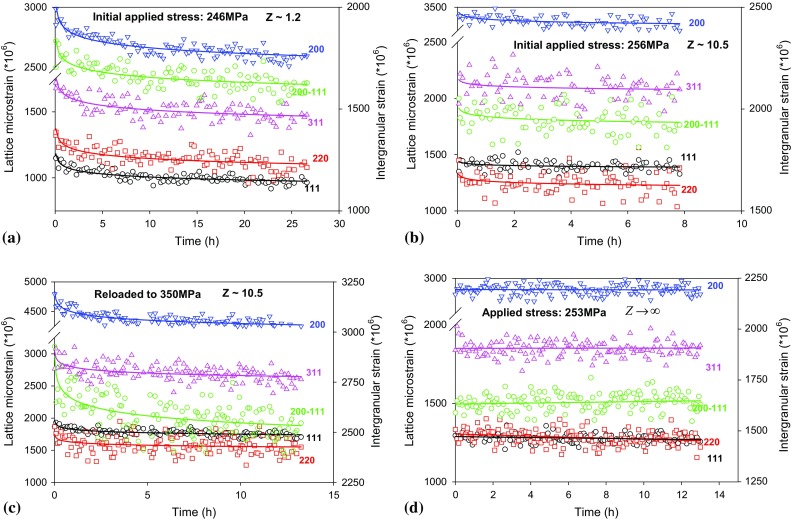
Elastic lattice strains and intergranular strain (between {200} and {111}) evolution measured in situ during early stage of creep in Type 316H austenitic stainless steel at 550 °C under **a** σ = 246 MPa for Z ~ 1; **b** σ = 256 MPa, Z ~ 10.5; **c** σ = 350 MPa, Z ~ 10.5; and **d** σ = 253 MPa, $Z \to \infty$. *Green circles* and *green lines* correspond to the secondary *Y* axis on the right

**Figure 4:**
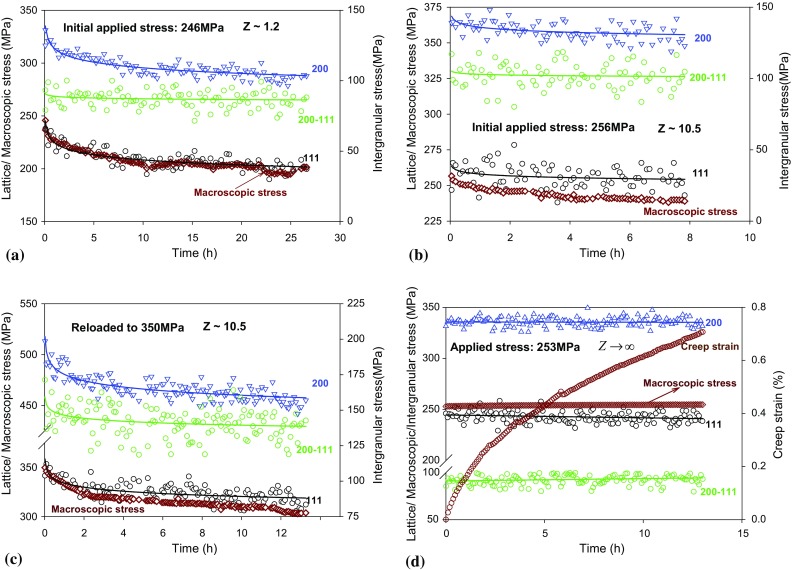
Lattice stresses and intergranular stress (between {200} and {111}) evolution under **a** σ = 246 MPa for Z ~ 1.2; **b** σ = 256 MPa, Z ~ 10.5; **c** σ = 350 MPa, Z ~ 10.5; and **d** σ = 253 MPa, $Z \to \infty$. The lattice stresses were calculated using the lattice strain (Fig. 3) multiplied by the corresponding diffraction elastic constants (Table 1)

The evolution of lattice strains and stresses with time in grain families {111}, {200}, {220} and {311} crystallographic planes during creep with *Z* = 1.2, *Z* = 10.5 and $Z \to \infty$ are shown in Figs. 3a–d and 4a–d. The evolution of intergranular strain and stress between {200} and {111} crystallographic planes is displayed using green colour in the corresponding figures with a secondary *Y* axis on the right. All of the curves were fitted using power law. Again it shows that the lattice strains relaxed the most (−423, −120 and −562 microstrain) in the softest lattice plane {200} and the least (−205, −49, and −216 microstrain) in the hardest lattice plane {111} under constant strain and elastic follow-up control (Fig. 3a–c). Figure 4a–b shows that the presence of elastic follow-up decreased the macroscopic as well as lattice stress relaxation significantly. For the creep tests with applied or initial applied stress of 250 MPa, same amount of lattice stress had relaxed at different crystal planes and the trends agree with the macroscopic stress relaxation. However, the intergranular stress seems to have changed with time when the elastic follow-up creep test was reloaded to a higher stress of 350 MPa (Fig. 4c). This opened an interesting future study for the current material. With a very high applied stress, the elastic follow-up might have significant effect on the evolution of intergranular stress during creep.

Figure 4d shows that the constant load creep strain rate decreased significantly in the early stages of primary creep. However, the lattice strains and stresses, intergranular strain and stress were almost constant under constant load control (Figs. 3d and 4d). This is not in agreement with the previous ex situ measurements (same material and testing conditions) conducted by Chen et al. [12] which showed that the lattice strains generated in the {200} and {220} grain families 180 h of creep (creep strain 0.92%) were approximately 440 microstrain and −275 microstrain. The discrepancies between the current and Chen’s work demonstrate that the ex situ measurements might not be able to show the evolution of lattice strain precisely. The scatter of lattice strain in neutron diffraction measurements for a single sample could be as large as 300 microstrain (Fig. 3d). Factors included sample-to-sample differences [20], difference of plastic strains created from loading up for each sample and thermal strains/stresses introduced in each sample during air quenching [21] can also change the lattice strains in materials significantly. The current in situ constant load creep results are also different to the in situ neutron diffraction creep experiments conducted by Rao et al. [11] in spite of similar macroscopic creep strain (~0.62%) generated from similar duration (12 h) of primary creep stage. Rao et al. [11] observed that the {200} grain family developed significant tensile creep strain (evolved from 0 to 850 microstrain) while the {111} and {220} developed compressive creep strains (evolved from 0 to −275 microstrain) during primary creep stage (180 MPa at 650 °C) in a solute heat-treated 316H austenitic stainless steel sample. This could be due to large number of carbides (10^21^ m^−3^) that can form along the grain boundaries, the slip planes and other entities within grain during the creep of the solution heat-treated sample at 650 °C [22]. The dislocation pinning therefore can change the internal resistance of each crystal plane [23], resulting in changing and redistribution of the lattice strain between grain families during creep deformation. Moreover, it is difficult to consider the change in stress-free lattice spacing due to solid solution carbon concentration [11].

## Conclusion

In conclusion, a pioneering study was conducted to investigate the effect of boundary conditions on the anisotropic creep behaviour along different crystal planes in Type 316H austenitic stainless steel. The presence of elastic follow-up decreased the macroscopic stress and lattice stress relaxation. With the initial applied stress of approximately 250 MPa at 550 °C, the lattice strains tended to relax the most in the softest lattice plane {200} and the least in the stiffest lattice plane {111} to maintain lattice stress equilibrium between different grain families. Unlike previous studies, we found under constant load control that both lattice strains and stresses remained constant. Therefore, the ratio of the current applied stress to intergranular stress decreases under constant strain and elastic follow-up control while it is constant under constant load control. Nevertheless, the present study shows that the intergranular strains or stresses are not the main reason cause to the decreasing of primary creep strain rate. For elastic follow-up creep test with higher applied stress (350 MPa), both the intergranular stresses and strains were changed. This indicated that the elastic follow-up might have an effect on the evolution of intergranular strains/stresses during creep with very high applied stress. The generation of intergranular stresses under creep is different or more complicated than under monotonic loading. The evolution of lattice strain in materials during creep can change due to their precipitation strengthening, boundary conditions as well as testing temperatures and stresses.
